# Theoretical study of population inversion in active doped MIR chalcogenide glass fibre lasers (invited)

**DOI:** 10.1007/s11082-014-0086-x

**Published:** 2014-12-11

**Authors:** S. Sujecki, A. Oladeji, A. Phillips, A. B. Seddon, T. M. Benson, H. Sakr, Z. Tang, E. Barney, D. Furniss, Ł. Sójka, E. Bereś-Pawlik, K. Scholle, S. Lamrini, P. Furberg

**Affiliations:** 1 Electrical Systems and Optics Division, George Green Institute for Electromagnetics Research, The University of Nottingham, University Park, Nottingham, NG7-2RD UK; 2 Institute of Telecommunications and Acoustics, Wrocław University of Technology, Wybrzeże Wyspiańskiego 27, 50-370 Wrocław, Poland; 3 LISA Laser Products OHG Fuhrberg & Teichmann, Max-Planck-Str. 1, 37191 Katlenburg-Lindau, Germany

**Keywords:** Fibre lasers, Low phonon energy glasses, Fibre laser modelling, Mid-infrared light

## Abstract

We study the mechanism of the population inversion in mid-infrared fibre lasers based on a chalcogenide glass host doped with active lanthanide ions. Three lanthanide dopant ions are considered: terbium, dysprosium and praseodymium. We predict the relevant trivalent ion level populations and gain. The simulation parameters were obtained by fabricating and optically characterising a series of trivalent ion doped chalcogenide glass samples. We also provide simple analytical expressions that aid the design of the cascade lasing process.

## Introduction

Mid-infrared (MIR) coherent light sources find application in medicine, environmental monitoring, the pharmaceutical industry and defence (Seddon 2011; Steinmeyer and Skibina 2014). Currently available MIR lasers include quantum cascade lasers (QCLs), interband cascade lasers (ICLs), optical parametric oscillators (OPOs), difference frequency generation (DFG) sources, solid state, fibre and gas lasers.

Lanthanide-doped fibre lasers are well established in the visible and near-infrared part of the optical spectrum. These sources rely on the classical three and four level pumping scheme . As far as the host glass is concerned, silica-glass fibres have dominated this application area due to their material robustness. However, for reaching wavelengths significantly longer than $2\,\upmu \hbox {m}$, other host glass materials need to be applied. This is necessary due to the large phonon energy of silica-glass. Wavelengths nearly up to $4\,\upmu \hbox {m}$ can be reached using a fluoride glass host. A holmium-doped fluoride glass fibre laser currently holds the record for the longest lasing wavelength of $3.9\,\upmu \hbox {m}$ (Schneider et al. 1997) obtained under liquid nitrogen cooling. In order to reach wavelengths beyond $4 \,\upmu \hbox {m}$, other glass hosts must be considered. So far, the most promising glass host for the realisation of longer wavelength MIR fibre lasers appears to be the chalcogenide glasses. The low phonon energy of these glasses, of down to about $250\hbox { cm}^{-1}$, potentially allows for the realisation of fibre lasers that operate well beyond the $4\,\upmu \hbox {m}$ barrier of the fluoride glasses. The chalcogenide glasses have been demonstrated independently by many research laboratories to effectively dissolve lanthanide ions (Shaw et al. 2001; Churbanov et al. 2003; Tang et al. 2011). Further, core-clad and micro-structured chalcogenide fibres with low losses at MIR wavelengths have been realised (Snopatin et al. 2009; El-Amraoui et al. 2010). Finally, core-clad, lanthanide-doped chalcogenide glass fibres (Sojka et al. 2014), including small-core (Tang et al. 2014) have been recently demonstrated various active lanthanide ions have been considered for the realisation of MIR fibre lasers (Shaw et al. 2001; Sojka et al. 2012). The most promising candidates for realising a MIR fibre laser include: dysprosium, terbium and praseodymium. In this study, we therefore theoretically investigate and discuss the possibility of the energy level population inversion in chalcogenide glass fibre lasers doped with $\hbox {Dy}^{3+}$, $\hbox {Pr}^{3+}$, and $\hbox {Tb}^{3+}$ ions. We also define the theoretical conditions of lasing in MIR spectral range.

## Theory

**Fig. 1:**
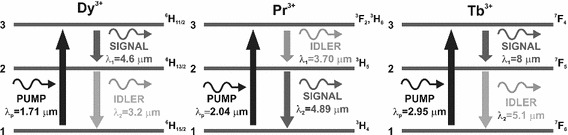
Simplified energy level diagram for isolated $\hbox {Dy}^{3+}$, $\hbox {Pr}^{3+}$ and $\hbox {Tb}^{3+}$

Figure 1 shows a simplified energy diagram of: (a) dysprosium; (b) praseodymium and (c) terbium trivalent ions. For the realisation of high efficiency MIR fibre lasers using $\hbox {Dy}^{3+}$, $\hbox {Pr}^{3+}$, and $\hbox {Tb}^{3+}$ doping, it is necessary to apply a cascade lasing system (Sojka et al. 2012). Unlike (Sojka et al. 2012), we nominally assume that in $\hbox {Tb}^{3+}$ the upper transition corresponds to the signal wave. It must be noted, however, that an efficient photoluminescence from this transition so far has not been experimentally observed (Shaw et al. 2001). In the case of $\hbox {Dy}^{3+}$ and $\hbox {Tb}^{3+}$, the idler wave depletes the lower lying energy level for the signal while in the case of $\hbox {Pr}^{3+}$ it mediates in populating the higher lying energy level. The standard laser configuration relies therefore on trapping the idler wave within the fibre laser cavity using either inscribed fibre gratings (Quimby et al. 2008) or simply Fresnel reflections at the fibre end faces (Sujecki et al. 2010).

In steady state, the level populations for energy systems shown in Fig. 1, in the absence of up-conversion phenomena, can be obtained by solving the following equations (Sujecki et al. 2010):

1 $$\begin{aligned} \left[ \begin{array}{l@{\quad }l@{\quad }l} a_{11}&{}a_{12}&{}a_{13}\\ a_{21}&{}a_{22}&{}a_{23}\\ 1&{} 1&{} 1\\ \end{array}\right] \times \left[ \begin{array}{l} N_1\\ N_2\\ N_3\\ \end{array}\right] =\left[ \begin{array}{l} 0\\ 0\\ N\\ \end{array}\right] \end{aligned}$$

In (1) the coefficients $\hbox {a}_\mathrm{xx}$ are as follows: $\hbox {a}_{11}=\sigma _\mathrm{pa} \times \upphi _\mathrm{p}$; $\hbox {a}_{12}=\sigma _{\lambda 1\mathrm{a}} \times \upphi (\lambda _{1})$; $\hbox {a}_{13} = -\sigma _\mathrm{pe} \times \upphi _\mathrm{p}-\sigma _{\lambda 1\mathrm{e}} \times \upphi (\lambda _{1})-1/\tau _{3}$; $\hbox {a}_{21}=\sigma _{\lambda 2\mathrm{a}} \times \upphi (\lambda _{2})$; $\hbox {a}_{22} = -\sigma _{\lambda 2\mathrm{e}} \times \upphi (\lambda _{2})-\sigma _{\lambda 1\mathrm{a}} \times \upphi (\lambda _{1})-1/\tau _{2}$; $\hbox {a}_{23}=\sigma _{\lambda 1\mathrm{e}} \times \upphi (\lambda _{1})+\upbeta _{32} \times \tau _{3}$ where $\tau _{3}$ and $\tau _{2}$ are the lifetimes of level 3 and 2, respectively, while $\upbeta _{32}$ is the branching ratio for the 3 -$>$ 2 transition and $\sigma _\mathrm{xya/e}$ is the absorption/emission cross-section for the xy transition. $\upphi _{p}$ is the value of the photon flux for the pump while $\upphi (\lambda _{1})$ and $\upphi (\lambda _{2})$ denote the photon fluxes of the waves interacting with levels 3–2 and 2–1, respectively.

## Results

**Fig. 2:**
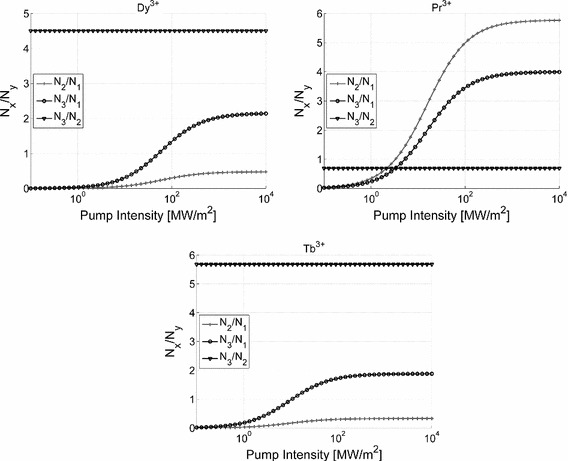
Dependence of population inversion ratios on the pump intensity for a chalcogenide GeAsGaSe glass doped with trivalent dysprosium, praseodymium and terbium ions. Simulation parameters are listed in Table 1

Figure 2 shows the dependence of the population inversion ratios on the pump intensity that were obtained by solving (1) in the absence of the idler and signal light. The values of the absorption and emission cross sections and the photoluminescence lifetimes were obtained experimentally. For this purpose we fabricated a series of GaAsGeSe chalcogenide bulk glass samples doped with $\hbox {Dy}^{3+}$, $\hbox {Pr}^{3+}$ and $\hbox {Tb}^{3+}$ ions, measured their optical absorption spectra using the Fourier Transform Infrared Spectroscopy and extracted the emission and absorption cross sections and the photoluminescence lifetimes following the procedure described in detail in (Sojka et al. 2012). The extracted simulation parameters are presented in Table 1.

**Table 1:** Trivalent lanthanide ion doped glass sample parameters used in calculations

Parameter	$\hbox {Dy}^{3+}$	$\hbox {Pr}^{3+}$	$\hbox {Tb}^{3+}$
$\upbeta _{32} \times \tau _{3}/\hbox {ms}$	27.5	6.9	67
$\tau _{21}/\hbox {ms}$	5.2	10.3	11.8
$\tau _{3}/\hbox {ms}$	2.2	2.7	5.9
$\sigma _{pa}/\hbox {m}^{2}$	$0.82 \times 10^{-24}$	$4 \times 10^{-24}$	$1 \times 10^{-24}$
$\sigma _\mathrm{pe}/\hbox {m}^{2}$	$0.38 \times 10^{-24}$	$1 \times 10^{-24}$	$0.53 \times 10^{-24}$
$\sigma _{\lambda 1\mathrm{a}}/\hbox {m}^{2}$	$0.3 \times 10^{-24}$	$1.03 \times 10^{-24}$	$0.55 \times 10^{-24}$
$\sigma _{\lambda 1\mathrm{e}}/\hbox {m}^{2}$	$0.44 \times 10^{-24}$	$1.24 \times 10^{-24}$	$0.75 \times 10^{-24}$
$\sigma _{\lambda 2\mathrm{a}}/\hbox {m}^{2}$	$0.037 \times 10^{-24}$	$0.68 \times 10^{-24}$	$0.42 \times 10^{-24}$
$\sigma _{\lambda 2\mathrm{e}}/\hbox {m}^{2}$	$0.15 \times 10^{-24}$	$0.86 \times 10^{-24}$	$0.7 \times 10^{-24}$

The results from Fig. 2 show that the inversion of population for the signal wave in $\hbox {Dy}^{3+}$ and $\hbox {Tb}^{3+}$ ion doped chalcogenide glass can be achieved at an arbitrarily low pump power. In fact the value of the $\hbox {N}_{3}/\hbox {N}_{2}$ ratio is equal exactly to $\upbeta _{32} \times \tau _{3}/\tau _{21}$, which can be shown by solving (1) analytically. Consequently the population inversion for the idler wave in the case of $\hbox {Pr}^{3+}$ doped glass cannot be achieved since $\upbeta _{32} \times \tau _{3} < \tau _{21}$.

In the case of the $\hbox {Pr}^{3+}$ ion, the inversion of population for the signal wave has to be achieved with respect to the ground state. This requires an incident pump flux of approximately $3.3\,\hbox {MW}/\hbox {m}^{2}$. Such power density levels at $2\,\upmu \hbox {m}$ wavelength, can however be effectively delivered by commercialy available laser diodes and fibre lasers, and are below the optical damage threshold for the chalcogenide glass, which is currently estimated at about $10\,\hbox { MW}/\hbox {m}^{2}$. In fact the exact value of the threshold pump intensity can be obtained by deriving first an analytical expression for the $\hbox {N}_{2}/\hbox {N}_{1}$ ratio from (1):

2 $$\begin{aligned} \frac{N_2 }{N_1 }=\frac{\frac{\sigma _{pa} }{\beta _{32} \tau _3 }\phi _p }{\frac{\sigma _{pe} }{\tau _{21} }\phi _p +\frac{1}{\tau _3 \tau _{21} }}\mathop \rightarrow \limits ^{\phi _p \rightarrow \infty } \frac{\sigma _{pa} \tau _{21} }{\sigma _{pe} \beta _{32} \tau _3 } \end{aligned}$$

Equating (2) to 1 yields the desired analytical expression for the threshold pump intensity:

3 $$\begin{aligned} I_{thr} =\frac{h\nu }{\tau _3 \tau _{21} \left( {\frac{\sigma _{pa} }{\beta _{32} \tau _3 }-\frac{\sigma _{pe} }{\tau _{21} }} \right) } \end{aligned}$$

Formula (3) shows that the threshold pump intensity can be effectively reduced by selecting the pumping wavelength at which the pump absorption cross-section is large. The values of the photoluminescence lifetimes, on the other hand, are fairly independent of the chalcogenide glass host composition for small compositional changes (Sakr et al. 2014; Seddon et al. 2010), hence their variations have limited impact on the threshold pump intensity. From (3) it follows that if $\upbeta _{32}\times \tau _{3} > >\tau _{21}$ then the population inversion is not possible, which is the case for the idler wave in $\hbox {Dy}^{3+}$ and $\hbox {Tb}^{3+}$ ion doped chalcodenide glass (c.f. Fig. 2 and Table 1).

**Fig. 3:**
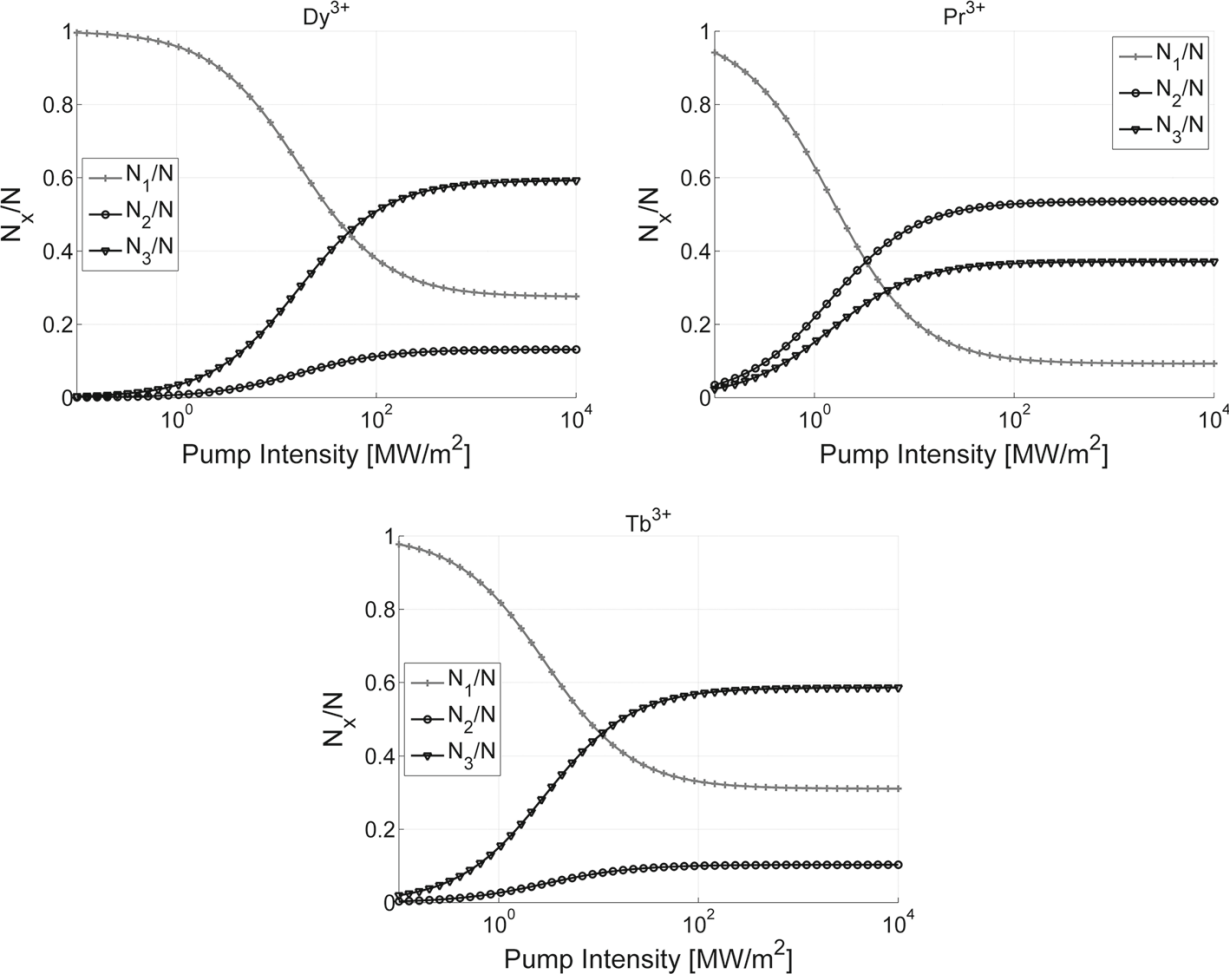
Dependence of level populations on the pump intensity for chalcogenide GeAsGaSe glass doped with trivalent dysprosium, praseodymium and terbium ions. Simulation parameters are listed in Table 1

Figure 3 shows the dependence of the level populations on the pump intensity in the absence of the idler and signal waves. Again the level populations at large values of the pump power are expressed via simple analytical formulae:

4 $$\begin{aligned} N_1 =\frac{\sigma _{pe}}{\sigma _{pa}}\frac{N}{D}; N_2 =\frac{\tau _{21}}{\beta _{32} \tau _3}\frac{N}{D}; N_3 =\frac{N}{D} \end{aligned}$$

where $D = 1 + \tau _{21}{\big /}\beta _{32}\tau _{3} + \sigma _{pe}{\big /}\sigma _{pa}$ Formulae (4) show that the population of the upper level for the signal transition is mainly dependent on the photoluminescence lifetimes, which are fairly fixed. The contribution of the emission and absorbtion cross-sections is relatively small, especially because the pump wavelength is selected so that $\sigma _\mathrm{pe} < \sigma _\mathrm{pa}$. Thereafter the maximum signal upper level populations of 0.5 N for $\hbox {Pr}^{3+}$ and 0.6 N for $\hbox {Dy}^{3+}$ and $\hbox {Tb}^{3+}$ are fairly indicative of the three considered lanthanide ions.

**Fig. 4:**
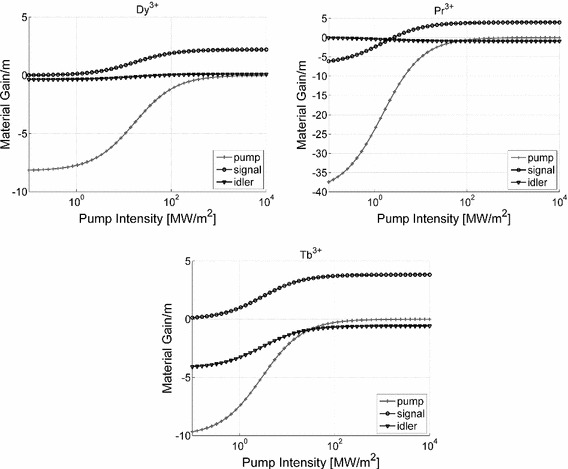
Dependence of level populations on the pump intensity for chalcogenide glass doped with trivalent dysprosium, praseodymium and terbium ions. Simulation parameters are listed in Table 1 and $\hbox {N} = 10^{25}/\hbox {m}^{3}$

Figure 4 shows the dependence of the material gain on the pump intensity, calculated in the absence of the idler and signal waves for $\hbox {N} = 10^{25}/\hbox {m}^{3}$, which in a GeAsGaSe chalcogenide glass corresponds approximately to 500 ppmw and is a typical value of the lanthanide ion concentration in a chalcogenide glass processed fibre that does not induce glass crystallisation (Tang et al. 2011). It is important to note that the curves shown in Figs. 2 and 3 do not depend on N since all level populations are proportional to N. Therefore, the gain values for other-ion concerntration levels can be extracted from Fig. 4 by simply scaling the results proportionally.

The results from Fig. 4 show that the maximum gain achieveable with terbium and praseodymium ions is approximately twice that available from dysprosium. Also, a high gain with the former two ions is available at lower values of the pump intensity when compared with the latter one. Further, the gain of 4/m potentially available with $10^{25}/\hbox {m}^{3}\,\hbox {Tb}^{3+}$ and $\hbox {Pr}^{3+}$ ions is sufficient to overcome fibre loss of up to 17.2 dB/m, which leaves a fair margin considering that a loss as low as 12 dB/km has been achieved in an undoped chalcogenide glass fibre (Snopatin et al. 2009).

## Conclusions

We performed a theoretical analysis of the population inversion mechanism in chalcogenide glasses doped with three selected trivalent lanthanide ions. The equations that describe the level populations admit simple analytical solutions that yield useful formulae for the pump threshold intensity calculation. The theoretical results obtained show that in $\hbox {Dy}^{3+}$ and $\hbox {Tb}^{3+}$ ion doped chalcogenide glass the population inversion for the signal wave can be achieved at lower values of pump power than in the case of $\hbox {Pr}^{3+}$. For $\hbox {Pr}^{3+}$ doped glass the theoretically predicted pump power threshold is approximately equal to $3.3\,\hbox { MW}/\hbox {m}^{2}$. We note that such power density is considerably less than the optical damage threshold for the chalcogenide glass, which is estimated at about $10\,\hbox { MW}/\hbox {m}^{2}$. Further, the theoretically achievable gain in $\hbox {Tb}^{3+}$ and $\hbox {Pr}^{3+}$ doped chalcogenide glass is approximately twice of that available from $\hbox {Dy}^{3+}$ doped chalcogenide glass and leaves a fairly large margin for compensating the intrinsic fibre loss.
